# Dynamic risk prediction models for different subtypes of hypertensive disorders in pregnancy

**DOI:** 10.3389/fsurg.2022.1005974

**Published:** 2022-10-26

**Authors:** Xinyu Zhang, Qi Xu, Lin Yang, Ge Sun, Guoli Liu, Cuiting Lian, Ziwei Li, Dongmei Hao, Yimin Yang, Xuwen Li

**Affiliations:** ^1^ Faculty of Environment and Life, Beijing University of Technology, Beijing, China; ^2^ Department of Obstetrics, Peking University People’s Hospital, Beijing, China

**Keywords:** hypertensive disorders in pregnancy, subtype, risk factor analysis, modeling method, dynamic prediction model, lasso regression

## Abstract

**Background:**

Hypertensive disorders in pregnancy (HDP) are diseases that coexist with pregnancy and hypertension. The pathogenesis of this disease is complex, and different physiological and pathological states can develop different subtypes of HDP.

**Objective:**

To investigate the predictive effects of different variable selection and modeling methods on four HDP subtypes: gestational hypertension, early-onset preeclampsia, late-onset preeclampsia, and chronic hypertension complicated with preeclampsia.

**Methods:**

This research was a retrospective study of pregnant women who attended antenatal care and labored at Beijing Maternity Hospital, Beijing Haidian District Maternal and Child Health Hospital, and Peking University People's Hospital. We extracted maternal demographic data and clinical characteristics for risk factor analysis and included gestational week as a parameter in this study. Finally, we developed a dynamic prediction model for HDP subtypes by nonlinear regression, support vector machine, stepwise regression, and Lasso regression methods.

**Results:**

The AUCs of the Lasso regression dynamic prediction model for each subtype were 0.910, 0.962, 0.859, and 0.955, respectively. The AUC of the Lasso regression dynamic prediction model was higher than those of the other three prediction models. The accuracy of the Lasso regression dynamic prediction model was above 85%, and the highest was close to 92%. For the four subgroups, the Lasso regression dynamic prediction model had the best comprehensive performance in clinical application. The placental growth factor was tested significant (*P* < 0.05) only in the stepwise regression dynamic prediction model for early-onset preeclampsia.

**Conclusion:**

The Lasso regression dynamic prediction model could accurately predict the risk of four HDP subtypes, which provided the appropriate guidance and basis for targeted prevention of adverse outcomes and improved clinical care.

## Introduction

Hypertensive disorders in pregnancy (HDP) are diseases that coexist with pregnancy and hypertension, which are major causes of increased maternal morbidity and mortality (1–3). HDP includes gestational hypertension, preeclampsia, eclampsia, chronic hypertension complicated with preeclampsia, and gestational combined chronic hypertension (4, 5). PE can be divided into two subtypes according to the time of onset: early-onset preeclampsia and late-onset preeclampsia (6, 7). HDP can be predicted by relevant risk factors, leading to early treatment (8–11).

The pathogenesis of HDP is complex. Risk factors for HDP are related to clinical epidemiological factors (12, 13), hemodynamic factors (14, 15), basic biochemical factors (16), and biomarkers (17, 18). For vascular biomarkers, numerous studies confirmed that placental growth factor (PlGF) had the function of regulating placental trophoblast and endothelial cells, and had a good predictive value for preeclampsia (19–21). HDP has multiple risk factors, which cannot be accurately predicted by a single factor and requires a combined assessment of multiple risk factors (22, 23). To improve the accuracy of prediction, researchers carried out a variety of combinations of different risk factors. Stepan et al. (24) found that a combination of ultrasound, mean arterial pressure, clinical features, and PlGF improved the prediction of preeclampsia in the first trimester of pregnancy. Chen et al. (25) found that the combination of mean arterial pressure, PlGF, and pregnancy-associated plasma protein A was far superior to a single factor. Current studies on the prediction of HDP focused on static studies at specific gestational weeks (26, 27), while pregnancy is a dynamic process and various physiological factors are constantly changing during pregnancy (28). Therefore, it is necessary to conduct a continuous dynamic study of HDP.

Different HDP subtypes are based on different physiological and pathological conditions of pregnant women, and a single modeling approach is not effective in predicting HDP subtypes. Poon et al. (29) found that the early-onset preeclampsia prediction model had a high detection rate of 93.1% for early-onset preeclampsia, but only 35.7% and 18.3% for late-onset preeclampsia and gestational hypertension. Sun et al. (30) compared the prediction effects of different methods on HDP and found that the Lasso regression method had the best prediction effect.

In this paper, we integrated multiple risk factors and multiple modeling approaches to develop dynamic prediction models for HDP subtypes. The prediction effects of various models were compared to select the optimal prediction model for effective prediction of each subtype.

## Materials and methods

### Research object

We performed a retrospective study on pregnant women who attended antenatal checkups at Beijing Maternity Hospital from 2006 to 2008, at Beijing Haidian District Maternal and Child Health Hospital from 2015 to 2016, and at Peking University People's Hospital from July 2015 to 2017. Our control group was healthy pregnant women without hypertensive disorders during pregnancy, not taking long-term medication, and without fetal malformations. A total of 1,267 women were included in this study, and they were divided into four HDP subgroups and a normal pregnancy group (Table 1).

**Table 1:** Subgroups of the studied population.

Group	Number of people
GH	205
EOPE	95
LOPE	234
CHCP	85
Control	648

GH, gestational hypertension; EOPE, early-onset preeclampsia; LOPE, late-onset preeclampsia; CHCP, chronic hypertension complicated with preeclampsia; Control, normal pregnancy women.

### Factors included in the analysis

The following data were collected from the maternal electronic medical records of the hospital: (1) the demographic data of pregnant women; (2) the clinical examination index. We classified the collected factors according to whether they changed with pregnancy: (a) static factors; (b) dynamic factors.

#### Static factors

Static factors were divided into two categories (Table 2): (i) quantitative factors, included age, height, and pre-pregnancy body mass index; (ii) qualitative factors, included first birth, multiple pregnancy, maternal history of disease, maternal family history of disease and maternal complications.

**Table 2:** Static factors.

Type	Factors
Quantitative factors	Age, height, pre-BMI
Qualitative factors	First birth, multiple pregnancy, history of spontaneous abortion, history of HDP, history of diabetes, family history of hypertension, family history of diabetes, gestational diabetes, pregestational diabetes mellitus, pregnancy combined with immune system disorders, pregnancy combined with hematologic disorders, pregnancy combined with thyroid disorders

Pre-BMI, pre-pregnancy body mass index.

#### Dynamic factors

Dynamic factors were divided into four categories (Table 3): (i) clinical epidemiologic factors; (ii) hemodynamic factors; (iii) basic biochemical factors; (iiii) biomarkers.

**Table 3:** Dynamic factors.

Type	Factors
Clinical epidemiologic factors	BMI
Hemodynamic factors	SBP, DBP, PP, MAP, K, CO, CI, TPR
Blood biochemical factors	HCT, MPV, PLT, ALT, AST, CRE, UA
Biomarkers	PlGF

BMI, body mass index; SBP, systolic blood pressure; DBP, diastolic blood pressure; PP, pulse pressure; MAP, mean arterial pressure; K, pulse wave shape coefficient; CO, cardiac output; CI, cardiac index; TPR, total peripheral resistance; HCT, hematocrit; MPV, mean platelet volume; PLT, platelet count; ALT, aspartame aminotransferase; AST, alanine aminotransferase; CRE, creatinine; UA, uric acid; PlGF, placental growth factor.

### Dynamic prediction model

In this paper, the data were characterized by a large variety of parameters and the data volume was a small sample (in thousands), so we chose nonlinear regression, support vector machine (SVM), stepwise regression and Lasso regression to develop the prediction models. The advantages of these methods were that they were suitable for small samples and had good generalization ability. Among these methods, stepwise regression and Lasso regression had the function of automatic filtering variables.

Dynamic factors changed continuously during pregnancy, so we included the gestational week as a parameter in this research from both the formula and algorithm perspectives: we constructed a custom regression dynamic gestational week fitting formula by using nonlinear regression; we developed dynamic prediction models by using SVM, stepwise regression and Lasso regression. In model training for each subgroup, we selected 15 pregnant women in the subgroup and control group to form the validation set, and the rest pregnant women were divided into training set and test set at a ratio of 7:3.

### Statistical analysis

Quantitative factors are presented as X (mean) ± SD (standard deviation). Qualitative factors are presented as percentages (%). Risk factors were screened for each HDP subgroup. For static factors, we conducted independent sample T test for quantitative factors and selected factors with *P* < 0.05; we performed chi-square test for qualitative factors, and selected factors with OR > 1 and *P* < 0.05. For dynamic factors, the clinical epidemiological factors and biomarkers for this research were body mass index and PlGF. A large number of researches had confirmed that body mass index and PlGF were risk factors for HDP (31, 32), so we analyzed the hemodynamic and basic biochemical factors. We divided the pregnant woman's gestational weeks into five stages: 0–13, 14–20, 21–28, 29–34, 35–40 weeks. We performed independent sample *t*-tests for hemodynamic and basic biochemical factors at each stage, and selected factors with *P* < 0.05 and abnormal value (mean value outside the normal range).

We used IBM SPSS Statistics 26.0 to develop a custom dynamic gestational week fitting formula. Matlab 2019b was used for SVM model research. R software (v4.0.1) was used for stepwise regression and Lasso regression model researches. We compared model prediction effects by area under the ROC curve (AUC), accuracy, and the model was externally validated by the validation set.

## Results

### Analysis of static risk factors

For gestational hypertension, we compared qualitative factors between the gestational hypertension group and the control group, and found there were statistically significant differences in multiple pregnancy, gestational diabetes and pregestational diabetes mellitus between the two groups (OR > 1 and *P* < 0.05). The pre-pregnancy body mass index of gestational hypertension group was significantly higher than that of control group (*P* < 0.05) (Table 4). For early-onset preeclampsia, the qualitative factors that met OR > 1 and *P* < 0.05 were multiple pregnancy, history of spontaneous abortion and history of HDP. The quantitative factors that met *P* < 0.05 was pre-pregnancy body mass index (Table 5). For late-onset preeclampsia, we found significant differences between the late-onset preeclampsia group and control group in multiple pregnancy, history of spontaneous abortion, history of HDP, family history of hypertension and family history of diabetes (OR > 1 and *P* < 0.05). The quantitative factors that were significantly different between the two groups was pre-pregnancy body mass index (*P* < 0.05) (Table 6). For chronic hypertension complicated with preeclampsia, qualitative factors of multiple pregnancy, family history of hypertension, gestational diabetes, pregestational diabetes mellitus, pregnancy combined with immune system disorders and pregnancy combined with thyroid disorders were risk factors of chronic hypertension complicated with preeclampsia (OR > 1 and *P* < 0.05). Pre-pregnancy body mass index among the quantitative factors was a risk factor for chronic hypertension combined with preeclampsia (*P* < 0.05) (Table 7).

**Table 4:** Static factors analysis of gestational hypertension.

Factor	GH	Control	OR
Qualitative factors			
First birth	145 (76.3%)	515 (81.4%)	0.738
Multiple pregnancy	5 (2.6%)*	4 (0.6%)	4.250
History of spontaneous abortion	47 (24.7%)	141 (22.3%)	1.147
History of HDP	1 (0.5%)	1 (0.2%)	3.344
History of diabetes	5 (2.6%)	10 (1.6%)	1.684
Family history of hypertension	41 (21.6%)	105 (16.6%)	1.384
Family history of diabetes	8 (4.2%)	32 (5.1%)	0.826
Gestational diabetes	21 (11.1%)*	37 (5.8%)	2.002
Pregestational diabetes mellitus	5 (2.6%)*	2 (0.3%)	8.527
Immune system disorders in pregnancy	4 (2.1%)	14 (2.2%)	0.951
Hematologic disorders in pregnancy	4 (2.1%)	20 (3.2%)	0.659
Thyroid disease in pregnancy	14 (7.4%)	30 (4.7%)	1.599
Quantitative factors			
Age (years)	30.830 ± 3.908^a^	30.220 ± 3.742^a^	–
Height (m)	1.626 ± 0.049^a^	1.624 ± 0.048^a^	–
Pre-BIM (kg/m^2^)	23.926 ± 4.503^a^^,^**	21.140 ± 3.101^a^	–

GH, gestational hypertension; Control, normal pregnancy women; Pre-BMI, pre-pregnancy body mass index.

^a^ Mean and standard deviation.

* *P* < 0.05 compared to Control.

** *P* < 0.05 compared to Control.

**Table 5:** Static factors analysis of early-onset preeclampsia.

Factor	EOPE	Control	OR
Qualitative factors			
First birth	56 (70.0%)	515 (81.4%)	0.535
Multiple pregnancy	7 (8.8%)**	4 (0.6%)	15.079
History of spontaneous abortion	39 (48.8%)**	141 (22.3%)	3.319
History of HDP	2 (2.5%)*	1 (0.2%)	16.205
History of diabetes	2 (2.5%)	10 (1.6%)	1.597
Family history of hypertension	15 (18.8%)	105 (16.6%)	1.160
Family history of diabetes	2 (2.5%)	32 (5.1%)	0.482
Gestational diabetes	2 (2.5%)	37 (5.8%)	0.413
Pregestational diabetes mellitus	0	2 (0.3%)	0.997
Immune system disorders in pregnancy	2 (2.5%)	14 (2.2%)	1.134
Hematologic disorders in pregnancy	2 (2.5%)	20 (3.2%)	0.786
Thyroid disease in pregnancy	2 (2.5%)	30 (4.7%)	0.515
Quantitative factors			
Age (years)	30.650 ± 4.543^a^	30.220 ± 3.742^a^	–
Height (m)	1.618 ± 0.051^a^	1.624 ± 0.048^a^	–
Pre-BIM (kg/m^2^)	55.734 ± 8.588^a^^,^**	21.140 ± 3.101^a^	–

EOPE, early-onset preeclampsia; control, normal pregnancy women; Pre-BMI, pre-pregnancy body mass index.

^a^ Mean and standard deviation.

* *P* < 0.05 compared to Control.

** *P* < 0.05 compared to Control.

**Table 6:** Static factors analysis of late-onset preeclampsia.

Factor	LOPE	Control	OR
Qualitative factors			
First birth	172 (78.5%)	515 (81.4%)	0.839
Multiple pregnancy	12 (5.5%)**	4 (0.6%)	9.116
History of spontaneous abortion	90 (41.1%)**	141 (22.3%)	2.434
History of HDP	6 (2.7%)**	1 (0.2%)	17.803
History of diabetes	7 (3.2%)	10 (1.6%)	2.057
Family history of hypertension	51 (23.3%)*	105 (16.6%)	1.527
Family history of diabetes	24 (11.0%)*	32 (5.1%)	2.312
Gestational diabetes	12 (5.5%)	37 (5.8%)	0.934
Pregestational diabetes mellitus	1 (0.5%)	2 (0.3%)	1.447
Immune system disorders in pregnancy	9 (4.1%)	14 (2.2%)	1.895
Hematologic disorders in pregnancy	2 (0.9%)	20 (3.2%)	0.282
Thyroid disease in pregnancy	8 (3.7%)	30 (4.7%)	0.762
Quantitative factors			
Age (years)	30.350 ± 4.300^a^	30.220 ± 3.742^a^	–
Height (m)	1.619 ± 0.053^a^	1.624 ± 0.048^a^	–
Pre-BIM (kg/m^2^)	23.239 ± 3.916^a^^,^**	21.140 ± 3.101^a^	–

LOPE, late-onset preeclampsia; Control, normal pregnancy women; Pre-BMI, pre-pregnancy body mass index.

^a^ Mean and standard deviation.

* *P* < 0.05 compared to Control.

** *P* < 0.05 compared to Control.

**Table 7:** Static factors analysis of chronic hypertension complicated with preeclampsia.

Factors	CHCP	Control	OR
Qualitative factors			
First birth	52 (74.3%)	515 (81.4%)	0.662
Multiple pregnancy	3 (4.3%)*	4 (0.6%)	7.041
History of spontaneous abortion	11 (15.7%)	141 (22.3%)	0.651
History of HDP	1 (1.4%)	1 (0.2%)	9.159
History of diabetes	0	10 (1.6%)	0.984
Family history of hypertension	21 (30.0%)*	105 (16.6%)	2.155
Family history of diabetes	3 (4.3%)	32 (5.1%)	0.841
Gestational diabetes	11 (15.7%)*	37 (5.8%)	3.003
Pregestational diabetes mellitus	6 (8.6%)**	2 (0.3%)	29.578
Immune system disorders in pregnancy	7 (10.0%)**	14 (2.2%)	4.913
Hematologic disorders in pregnancy	1 (1.4%)	20 (3.2%)	0.444
Thyroid disease in pregnancy	8 (11.4%)*	30 (4.7%)	2.594
Quantitative factors			
Age (years)	31.930 ± 5.123^a^^,^*	30.220 ± 3.742^a^	–
Height (m)	1.629 ± 0.055^a^	1.624 ± 0.048^a^	–
Pre-BIM (kg/m^2^)	24.142 ± 5.157^a^^,^**	21.140 ± 3.101^a^	–

CHCP, chronic hypertension complicated with preeclampsia; Control, normal pregnancy women; Pre-BMI, pre-pregnancy body mass index.

^a^ Mean and standard deviation.

* *P* < 0.05 compared to Control.

** *P* < 0.01 compared to Control.

### Analysis of dynamic risk factors

We analyzed all dynamic factors within the five gestational stages, and found dynamic factors were significantly different between the gestational hypertension group and the control group (Table 8). The difference in platelet count (PLT) between the early-onset preeclampsia group and the control group was not statistically significant, and the mean value did not exceed the normal range (Table 9). In this paper, we did not consider PLT as a risk factor for early-onset preeclampsia. We found there was no statistically significant differences in total peripheral resistance (TPR) between the late-onset preeclampsia group and the control group, but the TPR was outside the normal range at 10–13 and 35–40 weeks (Table 10). Therefore, we considered TPR as a risk factor for late-onset preeclampsia. The difference in pulse pressure (PP) between the chronic hypertension combined with preeclampsia group and the control group was not statistically significant, and the mean value did not exceed the normal range (Table 11). Therefore, we did not consider PP as a risk factor for chronic hypertension combined with preeclampsia.

**Table 8:** Dynamic factors analysis of gestational hypertension.

Factor	Group	10–13 weeks	14–20 weeks	21–27 weeks	28–34 weeks	35–40 weeks
SBP (mmHg)	GH	122.450*	120.556*	122.513*	125.667*	128.714*
	Control	115.581	111.809	109.199	110.033	109.543
DBP (mmHg)	GH	80.150*	77.917*	78.897*	79.190*	82.286*
	Control	73.806	69.953	68.460	69.510	69.139
PP (mmHg)	GH	42.300	42.639	43.615*	46.476*	46.429*
	Control	41.775	41.856	40.738	40.523	40.404
MAP (mmHg)	GH	95.544*	92.776*	95.436*	96.776*	100.645*
	Control	90.372	85.705	83.575	84.479	85.083
K	GH	0.375*	0.361*	0.386	0.379	0.398
	Control	0.402^#^	0.381	0.375	0.373	0.396
CO (L/min)	GH	4.824*	4.987*	4.783	5.475*	5.038*
	Control	4.316	4.784	4.871	4.967	4.493
CI [L/(min m^2^)]	GH	3.015	3.168*	2.884	3.147	2.867
	Control	2.778	3.043	3.006	3.000	2.633
TPR (mmHg s/ml)	GH	1.203^#^	1.077*	1.294^#^	1.096	1.267^#^
	Control	1.332^#^	1.139	1.090	1.080	1.231^#^
HCT (%)	GH	37.415	37.422*	36.793*	37.505*	37.368
	Control	37.599	35.222	35.183	36.119	36.509
MPV (fl)	GH	9.578*	10.686*	9.839	9.571	9.671
	Control	8.974	9.254	9.624	9.666	9.415
PLT (×10^9^/L)	GH	211.523	223.564	224.368*	198.786	202.536
	Control	223.024	220.193	205.692	196.365	196.159
ALT (U/L)	GH	17.162*	16.001*	20.319*	22.716	23.054
	Control	23.891	21.525	22.736	21.809	22.921
AST (U/L)	GH	20.096*	18.537*	21.190*	23.286	23.714
	Control	23.752	22.655	23.204	22.731	23.656
CRE (μmol/L)	GH	47.295*	52.869*	64.183	63.336*	55.543
	Control	52.284	61.909	65.824	49.658	55.100
UA (μmol/L)	GH	206.329	235.840*	245.833	282.490*	301.645*
	Control	200.676	240.863	246.089	228.416	265.853

SBP, systolic blood pressure; DBP, diastolic blood pressure; PP, pulse pressure; MAP, mean arterial pressure; K, pulse wave shape coefficient, dimensionless; CO, cardiac output; CI, cardiac index; TPR, total peripheral resistance; HCT, hematocrit; MPV, mean platelet volume; PLT, platelet count; ALT, aspartame aminotransferase; AST, alanine aminotransferase; CRE, creatinine; UA, uric acid.

* *P* < 0.05 compared to Control.

^#^ Value outside the normal range.

**Table 9:** Dynamic factors analysis of early-onset preeclampsia.

Factor	Group	10–13 weeks	14–20 weeks	21–27 weeks	28–34 weeks	35–40 weeks
SBP (mmHg)	EOPE	114.438	124.286*	120.000*	118.385*	148.963*^,^^#^
	Control	115.547	112.313	109.252	108.252	110.013
DBP (mmHg)	EOPE	75.438	77.857*	77.316*	75.538*	96.074*^,^^#^
	Control	73.795	70.270	68.454	68.034	69.479
PP (mmHg)	EOPE	39.000	46.429	42.684	42.846	52.889*^,^^#^
	Control	41.752	42.043	40.797	40.218	40.534
MAP (mmHg)	EOPE	91.553	95.680*	93.128*	91.603*	117.952*
	Control	90.336	86.029	83.583	84.195	84.453
K	EOPE	0.414^#^	0.387	0.373	0.385*	0.415*^,^^#^
	Control	0.401^#^	0.380	0.375	0.373	0.403^#^
CO (L/min)	EOPE	4.071	5.286	5.181	5.215	5.273*
	Control	4.320	4.795	4.876	4.969	4.302
CI [L/(min m^2^)]	EOPE	2.573	3.088	3.203	3.174	2.960*
	Control	2.781	3.048	3.006	3.000	2.547
TPR (mmHg s/ml)	EOPE	1.494^#^	1.121^#^	1.112	1.410*^,^^#^	1.105
	Control	1.331^#^	1.136^#^	1.088	1.079	1.267^#^
HCT (%)	EOPE	37.321	38.512*	38.329*	35.985	37.654
	Control	37.591	35.246	35.197	36.129	36.175
MPV (fl)	EOPE	8.902	9.106	9.692	9.678	10.477*
	Control	8.986	9.277	9.615	9.664	9.203
PLT (×10^9^/L)	EOPE	228.482	241.275	192.046	180.378	180.923
	Control	222.409	221.554	205.166	196.200	199.134
ALT (U/L)	EOPE	19.669	20.446	21.886	23.333*	23.500
	Control	23.786	21.423	22.692	21.814	22.765
AST (U/L)	EOPE	21.206	21.964	22.694	23.889*	24.000
	Control	23.710	22.497	23.175	22.735	23.563
CRE (μmol/L)	EOPE	47.761	62.517	62.098	76.275*	56.395
	Control	52.227	61.248	65.818	49.743	54.863
UA (μmol/L)	EOPE	212.681	231.447	232.684*	335.053*	276.247
	Control	200.542	240.577	246.082	228.289	263.255

SBP, systolic blood pressure; DBP, diastolic blood pressure; PP, pulse pressure; MAP, mean arterial pressure; K, pulse wave shape coefficient, dimensionless; CO, cardiac output; CI, cardiac index; TPR, total peripheral resistance; HCT, hematocrit; MPV, mean platelet volume; PLT, platelet count; ALT, aspartame aminotransferase; AST, alanine aminotransferase; CRE, creatinine; UA, uric acid.

* *P* < 0.05 compared to Control.

^#^ Value outside the normal range.

**Table 10:** Dynamic factors analysis of late-onset preeclampsia.

Factor	Group	10–13 weeks	14–20 weeks	21–27 weeks	28–34 weeks	35–40 weeks
SBP (mmHg)	LOPE	114.118	120.226*	120.039*	127.897*	136.083*
	Control	115.575	112.009	109.117	110.067	109.280
DBP (mmHg)	LOPE	73.647	76.547*	74.471*	80.971*	90.861*^,^^#^
	Control	73.856	70.164	68.400	69.537	68.986
PP (mmHg)	LOPE	40.471	43.679	45.569*	46.926*	45.222*
	Control	41.719	41.845	40.717	40.530	40.294
MAP (mmHg)	LOPE	90.119	92.526*	91.526*	98.891*	108.468*
	Control	90.369	85.847	83.510	84.510	84.928
K	LOPE	0.407^#^	0.374	0.379	0.383*	0.387
	Control	0.401^#^	0.380	0.375	0.373	0.397
CO (L/min)	LOPE	4.313	4.900	5.248	5.424*	5.206*
	Control	4.322	4.796	4.867	4.968	4.458
CI [L/(min m^2^)]	LOPE	2.774	3.183	3.195	3.201*	2.927*
	Control	2.782	3.055	3.005	3.003	2.627
TPR (mmHg s/ml)	LOPE	1.347^#^	1.149	1.118	1.152	1.339^#^
	Control	1.331^#^	1.134	1.090	1.080	1.238^#^
HCT (%)	LOPE	37.584	36.492*	36.633*	36.976	36.972
	Control	37.588	35.196	35.173	36.079	36.586
MPV (fl)	LOPE	8.943	9.710*	9.403	9.791	10.297*
	Control	8.987	9.285	9.600	9.660	9.411
PLT (×10^9^/L)	LOPE	223.983	201.779*	203.542	193.047	179.028
	Control	222.049	220.816	205.503	195.800	194.364
ALT (U/L)	LOPE	19.394	18.746*	22.673	22.334	23.500
	Control	23.788	21.506	22.769	21.834	22.888
AST (U/L)	LOPE	20.782	19.882*	22.845	23.326	24.000
	Control	23.708	22.572	23.215	22.754	23.636
CRE (μmol/L)	LOPE	48.396	53.677*	65.454	65.037*	61.924*
	Control	52.211	61.427	65.848	49.630	55.067
UA (μmol/L)	LOPE	203.576	211.578*	238.838*	304.248*	308.173*
	Control	200.624	240.662	246.135	228.304	265.950

SBP, systolic blood pressure; DBP, diastolic blood pressure; PP, pulse pressure; MAP, mean arterial pressure; K, pulse wave shape coefficient, dimensionless; CO, cardiac output; CI, cardiac index; TPR, total peripheral resistance; HCT, hematocrit; MPV, mean platelet volume; PLT, platelet count; ALT, aspartame aminotransferase; AST, alanine aminotransferase; CRE, creatinine; UA, uric acid.

* *P* < 0.05 compared to Control.

^#^ Value outside the normal range.

**Table 11:** Dynamic factors analysis of chronic hypertension complicated with preeclampsia.

Factors	Group	10–13 weeks	14–20 weeks	21–27 weeks	28–34 weeks	35–40 weeks
SBP (mmHg)	LOPE	125.429*	130.750*	136.091*	136.121*	136.167*
	Control	115.547	112.215	109.366	110.162	112.211
DBP (mmHg)	LOPE	81.810*	87.500*	92.455*^,^^#^	92.467*^,^^#^	92.533*^,^^#^
	Control	73.795	70.256	68.201	69.595	70.566
PP (mmHg)	LOPE	43.619	43.250	43.636	42.133	44.833
	Control	41.752	41.959	41.166	40.566	41.645
MAP (mmHg)	LOPE	96.579*	101.917*	107.244*	95.182*	108.871
	Control	90.336	86.109	83.496	84.582	87.043
K	LOPE	0.354*	0.350*	0.356*	0.370	0.392
	Control	0.401^#^	0.383	0.375	0.373	0.397
CO (L/min)	LOPE	4.981*	5.000*	4.817	5.044	4.951
	Control	4.320	4.746	4.908	4.981	4.627
CI [L/(min m^2^)]	LOPE	3.237*	3.250*	3.115	3.072	2.870
	Control	2.781	3.021	3.025	3.001	2.691
TPR (mmHg s/ml)	LOPE	1.068*	1.050*	1.121	1.173	1.342^#^
	Control	1.331^#^	1.151	1.081	1.077	1.224^#^
HCT (%)	LOPE	35.617*	37.124*	37.442*	37.173	38.767
	Control	37.585	35.258	35.206	36.317	36.343
MPV (fl)	LOPE	10.066*	11.312*	11.054*	10.600*	10.750*
	Control	8.986	9.226	9.596	9.725	9.523
PLT (×10^9^/L)	LOPE	195.799*	247.307	221.002	210.400	192.667
	Control	222.409	223.213	204.596	195.338	195.494
ALT (U/L)	LOPE	14.901*	28.181*	22.676	19.767	22.652
	Control	23.786	21.579	22.735	21.916	23.170
AST (U/L)	LOPE	24.147	50.114*^,^^#^	22.457	21.333	22.409
	Control	23.710	22.604	23.227	22.798	23.804
CRE (μmol/L)	LOPE	44.370*	59.639	52.385*	63.622*	62.585*
	Control	52.227	61.668	66.185	49.830	55.701
UA (μmol/L)	LOPE	220.557*	321.105*	252.957	302.115*	344.232*
	Control	200.542	240.701	246.347	228.714	264.888

SBP, systolic blood pressure; DBP, diastolic blood pressure; PP, pulse pressure; MAP, mean arterial pressure; K, pulse wave shape coefficient, dimensionless; CO, cardiac output; CI, cardiac index; TPR, total peripheral resistance; HCT, hematocrit; MPV, mean platelet volume; PLT, platelet count; ALT, aspartame aminotransferase; AST, alanine aminotransferase; CRE, creatinine; UA, uric acid.

* *P* < 0.05 compared to Control.

^#^ Value outside the normal range.

### Model construction results

**Figure 1:**
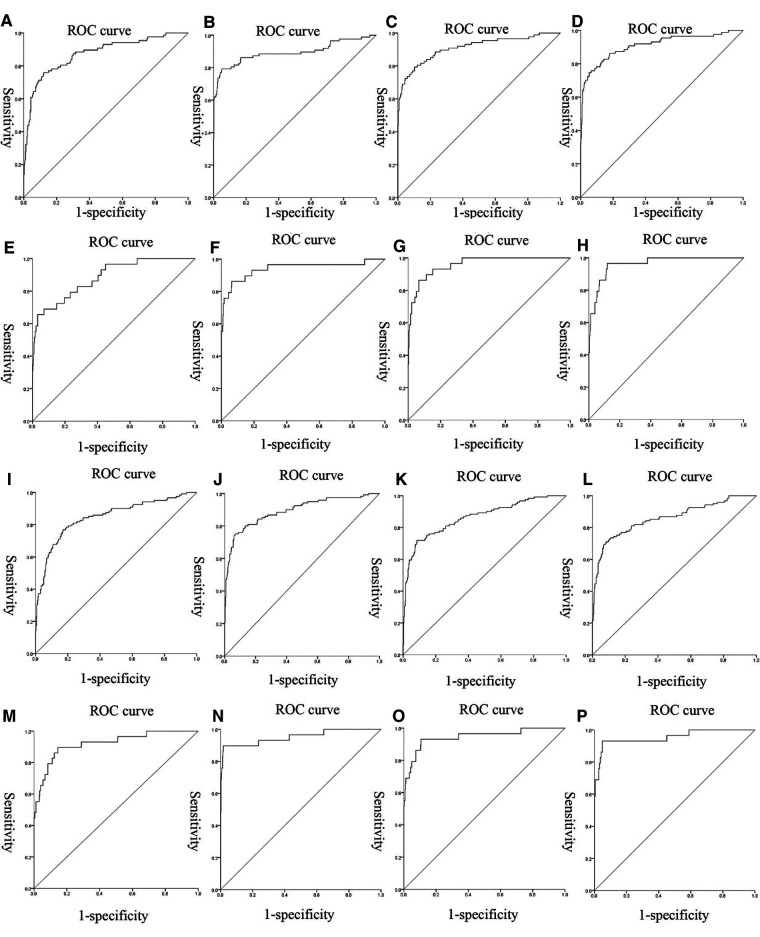
ROC curves of the models: (**A–D**) were the ROC curves of the four models of gestational hypertension; (**E–H**) were the ROC curves of the four models of early-onset preeclampsia; (**I–L**) were the ROC curves of the four models of early-onset preeclampsia; (**M–P**) were the ROC curves of the four models of chronic hypertension complicated with preeclampsia.

We used nonlinear regression, SVM, step regression and Lasso regression for each HDP subgroup to develop prediction models. The *P*-values of the models were all less than 0.001, which indicated that the models were stable. We compared the prediction results of the four models, the Lasso regression prediction model of the gestational hypertension was optimal: accuracy = 90.32%, AUC = 0.910, sensitivity = 75.86%, specificity = 93.32%; the Lasso regression prediction model of the early-onset preeclampsia was optimal: accuracy = 91.78%, AUC = 0.962, sensitivity = 86.21%, specificity = 92.18%; Lasso regression prediction model for late-onset preeclampsia was optimal: accuracy = 85.58%, AUC = 0.859, sensitivity = 72.73%, specificity = 89.47%; Lasso regression prediction model for chronic hypertension complicated with preeclampsia was optimal: accuracy = 91.72%, AUC = 0.955, sensitivity = 93.10%, specificity = 91.63% (Figure 1 and Table 12). PlGF was tested significant (*P* < 0.05) only in the stepwise regression dynamic prediction model for early-onset preeclampsia (Table 13), the predictive effect of PlGF in gestational hypertension, late-onset preeclampsia, and chronic hypertension complicated with preeclampsia was not significant, with parameter term coefficients of −3.26E−03, −1.39E−04, and −6.11E−03, respectively.

**Table 12:** Test results of the models.

Group	Model	*P*	AC (%)	SE (%)	SP (%)	AUC (95% CI)
GH	NLR	<0.001	79.25	79.31	79.24	0.873 (0.828–0.918)
	SVM	<0.001	93.08	72.41	97.37	0.894 (0.844–0.944)
	Step	<0.001	89.13	74.71	92.12	0.910 (0.870–0.951)
	Lasso	<0.001	90.32	75.86	93.32	0.910 (0.870–0.951)
EOPE	NLR	<0.001	77.40	75.86	77.51	0.884 (0.818–0.950)
	SVM	<0.001	95.66	75.86	97.07	0.940 (0.879–1.000)
	Step	<0.001	90.64	86.21	90.95	0.959 (0.929–0.989)
	Lasso	<0.001	91.78	86.21	92.18	0.962 (0.934–0.991)
LOPE	NLR	<0.001	80.38	76.86	81.45	0.847 (0.803–0.891)
	SVM	<0.001	88.27	62.81	95.99	0.894 (0.857–0.931)
	Step	<0.001	85.19	71.90	89.22	0.863 (0.822–0.905)
	Lasso	<0.001	85.58	72.73	89.47	0.859 (0.816–0.903)
CHCP	NLR	<0.001	83.91	89.66	83.50	0.921 (0.863–0.979)
	SVM	<0.001	97.70	75.86	99.26	0.952 (0.900–1.000)
	Step	<0.001	93.33	79.31	94.33	0.945 (0.893–0.998)
	Lasso	<0.001	91.72	93.10	91.63	0.955 (0.906–1.000)

NLR, nonlinear regression; SVM, support vector machine; Step, stepwise regression; Lasso, Lasso regression; AC, accuracy; SE, sensitivity; SP, sensitivity; AUC, area under the ROC curve.

**Table 13:** Parameters of the stepwise regression dynamic prediction model for the early-onset preeclampsia.

Factor	Coefficient
Gestational weeks	−1.12E−01*
Multiple pregnancy	3.25E+00**
History of spontaneous abortion	8.05E−01*
BMI	3.53E−01**
MAP	9.99E−02**
CO	−6.25E−01
CI	1.22E+00*
PlGF	−1.47E−02*
AST	−1.59E−01*
UA	1.66E−02**
Constant term	−1.79E+01**

BMI, body mass index; MAP, mean arterial pressure; CO, cardiac output; CI, cardiac index; PlGF, placental growth factor; AST, alanine aminotransferase; UA, uric acid.

* *P* < 0.05.

** *P* < 0.01.

The validation results showed that Lasso regression prediction model had the highest accuracy among the four prediction models in the chronic hypertension complicated with preeclampsia (Table 14).

**Table 14:** Validation results of the prediction models.

Group	Model	AC (%)	SE (%)	SP (%)
GH	NLR	93.33	100.00	86.67
	SVM	83.33	66.67	100.00
	Step	86.67	73.33	100.00
	Lasso	86.67	73.33	100.00
EOPE	NLR	80.00	93.33	66.67
	SVM	73.33	46.67	100.00
	Step	96.67	93.33	100.00
	Lasso	83.33	66.67	100.00
LOPE	NLR	80.00	73.33	86.67
	SVM	66.67	100.00	33.33
	Step	73.33	53.33	93.33
	Lasso	76.67	53.33	100.00
CHCP	NLR	83.33	100.00	66.67
	SVM	66.67	33.33	100.00
	Step	100.00	100.00	100.00
	Lasso	100.00	100.00	100.00

NLR, nonlinear regression; SVM, support vector machine; Step, stepwise regression; Lasso, Lasso regression; AC, accuracy; SE, sensitivity; SP, sensitivity.

## Discussion

Hypertensive pregnancy in disorders are pregnancy-specific systematic disorders that globally affect 5%–10% of all pregnancies (33, 34). We performed a comprehensive screening of high-risk factors for gestational hypertension, early-onset preeclampsia, late-onset preeclampsia and chronic hypertension combined with preeclampsia, which through the acquisition of clinical medical records of patients. For each HDP subtype, we constructed dynamic prediction models using nonlinear regression, support vector machines, stepwise regression, and Lasso regression. The results showed that the Lasso regression dynamic prediction model had the best prediction effect for the four HDP subtypes, which could help clinicians accurately assess the risk of HDP.

We compared the AUC of the four prediction models for each HDP subgroup, and we found that the AUC of the Lasso regression prediction model was higher than the other three prediction models. The accuracy of Lasso regression prediction model was over 85% for each HDP subgroup, and 91.78% for EOPE subgroup was the highest (Table 12). External validation of the model through the validation set, we found that Lasso regression prediction model had a good identification effect, with the accuracy of 86.67%, 83.33%, 76.67% and 100.00% for each HDP subtype, respectively (Table 14). Lasso regression allows automatic filtering of model parameters, and the Lasso regression model simplifies the input parameters of the model and makes the model structure simpler (Table 13).

PlGF is a member of the vascular endothelial growth factor family and has important functions in regulating placental trophoblast and endothelial cell function (35). Numerous studies have shown that PlGF is a risk factor for HDP and has a predictive value for preeclampsia in particular (36, 37). PlGF was tested significant only in the stepwise regression model for the early-onset preeclampsia, which indicated a significant predictive effect of PlGF on the early-onset preeclampsia (Table 13).

Finally, there were some limitations in this research. First, this research was carried out in China, and the medical records used for model construction were all from pregnant Chinese women. Due to differences among regions and races, the applicability of the model to other countries needs to be further verified. Second, we developed prediction models for the four HDP subtypes in this study and found that the lasso regression prediction model had the best prediction effect, so it was impossible to explore the predictive ability of other HDP subtype.

## Conclusion

We investigated the predictive effect of different variable selection and modeling approaches on HDP subtypes, and found the Lasso regression prediction model performed well and accurately predicted the risk of HDP subtypes. The Lasso regression prediction model provided corresponding guidance and served as a basis for preventing adverse outcomes and improving clinical treatment.

## Data Availability

The original contributions presented in the study are included in the article/Supplementary Material, further inquiries can be directed to the corresponding author/s.
